# Standardization of methods to record Vagus nerve activity in mice

**DOI:** 10.1186/s42234-018-0002-y

**Published:** 2018-03-15

**Authors:** Harold A. Silverman, Andrew Stiegler, Téa Tsaava, Justin Newman, Benjamin E. Steinberg, Emily Battinelli Masi, Sergio Robbiati, Chad Bouton, Patricio T. Huerta, Sangeeta S. Chavan, Kevin J. Tracey

**Affiliations:** 1 Center for Biomedical Sciences, Feinstein Institute for Medical Research, Northwell Health, 350 Community Drive, Manhasset, NY 11030 USA; 2 Hofstra Northwell Health School of Medicine, 350 Community Drive, Manhasset, NY 11030 USA; 3 Circulatory Technologies, Inc., 350 Community Drive, Manhasset, NY 11030 USA; 4 Department of Anesthesia, University of Toronto, 150 College Street, Toronto, ON M5S 3E2 Canada; 5 Laboratory of Immune & Neural Networks, Feinstein Institute for Medical Research, Northwell Health, 350 Community Drive, Manhasset, NY 11030 USA; 6 Center for Bioelectronic Medicine, Feinstein Institute for Medical Research, Northwell Health, 350 Community Drive, Manhasset, NY 11030 USA

**Keywords:** Vagus nerve recording, Neurogram, Murine, Inflammation, TLR4KO

## Abstract

**Background:**

The vagus nerve plays an important role in the regulation of organ function, including reflex pathways that regulate immunity and inflammation. Recent studies using genetically modified mice have improved our understanding of molecular mechanisms in the neural control of immunity. However, mapping neural signals transmitted in the vagus nerve in mice has been limited by technical challenges. Here, we have standardized an experimental protocol to record compound action potentials transmitted in the vagus nerve.

**Methods:**

The vagus nerve was isolated in Balb/c and B6.129S mice, and placed either on a hook or cuff electrode. The electrical signals from the vagus nerve were digitized using either a Neuralynx or Plexon data acquisition system. Changes in the vagus nerve activity in response to anesthesia, feeding and administration of bacterial endotoxin were analyzed.

**Results:**

We have developed an electrophysiological recording system to record compound action potentials from the cervical vagus nerve in mice. Cuff electrodes significantly reduce background noise and increase the signal to noise ratio as compared to hook electrodes. Baseline vagus nerve activity varies in response to anesthesia depth and food intake. Analysis of vagus neurograms in different mouse strains (Balb/c and C57BL/6) reveal no significant differences in baseline activity. Importantly, vagus neurogramactivity in wild type and TLR4 receptor knock out mice exhibits receptor dependency of endotoxin mediated signals.

**Conclusions:**

These methods for recording vagus neurogram in mice provide a useful tool to further delineate the role of vagus neural pathways in a standardized murine disease model.

## Background

Neural reflex circuits maintain physiological homeostasis by regulating the function of organ systems. Recent advances in neuroscience and immunology have revealed that neural reflexes also provide functional control over immune responses. This neural mediated immune regulation has evolutionary origin in worms with primitive neural and immune systems (Tracey, 2002; Andersson & Tracey, 2012; Styer et al., 2008). We have previously mapped a neural circuit, termed “the inflammatory reflex”, that is activated during infection, inflammation and injury when increasing levels of inflammatory mediators are sensed by the afferent vagus nerve (Tracey, 2002; Andersson & Tracey, 2012). The ascending information is relayed to the brainstem; and the resulting efferent response is mediated by the vagus nerve to the spleen and other organs (Rosas-Ballina et al., 2008). In spleen, these neural signals terminate on acetylcholine producing T cells (TChAT) to release acetylcholine (Rosas-Ballina et al., 2011). Binding of acetylcholine to its cognate receptor, α-7 nicotinic acetylcholine receptor (α7nAChR), on cytokine producing cells inhibits nuclear translocation of NF-kB and inflammasome activation, and suppresses cytokine production (Wang et al., 2004; Lu et al., 2014). Activation of the inflammatory reflex by direct electrical stimulation of the vagus nerve significantly attenuates cytokine release and ameliorates inflammation-mediated injury in endotoxemia, sepsis, colitis, and pre-clinical animal models of inflammatory diseases (Borovikova et al., 2000; Huston et al., 2008; van Westerloo & Giebelen, 2005; Ghia et al., 2006; Bernik et al., 2002; Levine et al., 2014). Recent clinical studies in patients with rheumatoid arthritis and Crohn’s disease indicate that stimulation of the inflammatory reflex significantly improves disease activity scores (Koopman et al., 2016; Bonaz et al., 2016).

Prior work demonstrated that afferent vagus nerve fibers play an important role in immune-to-brain communication (Tracey, 2002). Sub-diaphragmatic vagotomy prevents fever and sickness behavior after intraperitoneal administration of either cytokine interleukin-1β (IL1β) or lipopolysaccharide (LPS) (Watkins et al., 2015; Watkins et al., 1995; Watkins et al., 1994; Milligan et al., 1997; Gaykema et al., 1995; Hansen & Krueger, 1997). Further, binding of IL1β to glomus cells of vagus paraganglia results in activation of afferent vagus nerve signals (Goehler et al., 1997; Goehler et al., 1999). Intraperitoneal IL1β or LPS administration induce the expression of the activation marker c-Fos in vagal primary afferent neurons, indicating that cytokines activate vagus afferents and relay this information to the brain (Goehler et al., 1998; Gaykema et al., n.d.). Direct electrophysiogical recordings of compound action potentials in the vagus nerve have identified afferent and efferent activity following administration of IL1 (Niijima et al., 1991). Intraportal administration of IL1β increases the afferent activity in the hepatic vagus nerve, and reflex activation of efferent activity in the splenic nerve (Niijima, 1996). Hepatic vagotomy inhibits this reflex activation of the splenic nerve indicating that the hepatic vagus stimulated by IL1β in the portal venous blood initiates a reflex regulation of the splenic nerve (Niijima, 1996; Niijima et al., 1995). Together, these studies indicate that the vagus nerve responds to cytokines and transmits that information to the brainstem.

To date, development of neural recording techniques for rodent research has been limited. Here, we have developed an electrophysiological recording system to record compound action potentials (CAPs) from the cervical vagus nerve in mice and observed compound neurograms that vary in response to anesthesia, feeding, and administration of bacterial endotoxin (LPS).

## Methods

### Animals

All experimental protocols were approved by the Institutional Animal Care and Use Committee (IACUC) at the Feinstein Institute for Medical Research, Northwell Health, which follows the NIH guidelines for ethical treatment of animals. Male Balb/c and B6.129S mice were purchased from Charles River and Jackson Labs and used between 8 and 16 weeks of age. TLR4 knockout mice (TLR4 KO) were bred at the Feinstein Institute for Medical Research and used between the same age range. Mice were housed under reverse day/light cycle and had access to food and water ad libitum. In studies examining effect of food intake on vagus nerve activity, food was withheld for the 3–4 h prior to nerve recording; animals continued to have access to water.

### Electrode fabrication

**Fig. 1:**
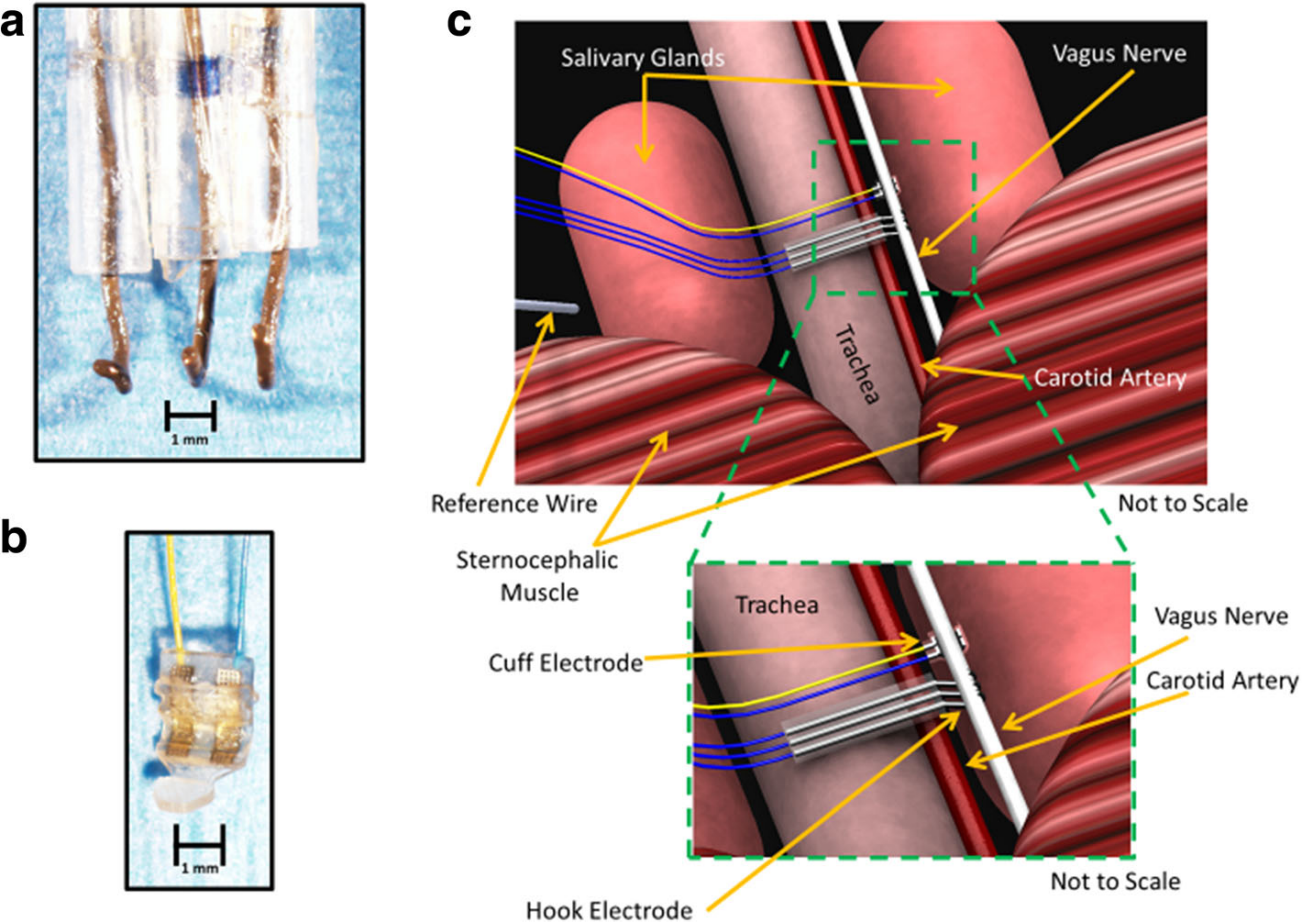
Electrophysiological recording system for the cervical vagus nerve. **a** Lab-made hook electrode with three leads was used to record cervical vagus nerve activity. **b** A cuff electrode purchased from Cortec was used to record cervical vagus nerve activity. **c** Artist rendering of isolated cervical vagus nerve (not to scale)

The hook electrodes with three leads were fabricated in-house (Fig. 1a). The hook electrode was fabricated with three 6.5 cm (hooks) and one 8 cm piece (ground) of 0.38 mm of silver wires soldered to the EIB. Prior to use, the hook electrode along with the ground were cleaned with bleach for 1 h to corrode the silver, and improve the contact with the nerve. In between experiments, the electrodes were cleaned with either 1 N hydrochloric acid or bleach. Impedance level of approximately ≤50kΩ was recorded in the hook electrode. The cuff electrodes with two leads were purchased from CorTec GmbH (Fig. 1b). Prior to use, the cuff was submerged in ethanol to both clean and strengthen the flexible outer material as per the manufacturer’s instructions. In between experiments, the cuff was cleaned with either saline or ethanol. The cuff electrode showed an impedance level of ≤150kΩ. Each electrode was connected to the electrode interface board (EIB), with slots for both the electrode and ground wire connections. The EIB then serially interfaced with the acquisition system through a head stage.

### Surgical isolation of cervical vagus nerve

Mice were induced in a supine position with general anesthesia using isoflurane at 2.5% in 100% oxygen, and maintained at 2.0% isoflurane during surgery. Once the nerve was isolated, anesthesia was maintained at approximately 1.75% during recordings for BALB/c mice and approximately 1.25% for B6.129S mice. For experiments comparing effects of different doses of isoflurane, the levels were maintained at either 2.0% or 1.75% or 1.5% in different groups of animals during recordings. The core body temperature was monitored with a rectal probe and maintained around 37 °C with a heating pad and heat lamp. To expose the cervical vagus nerve, the neck area was shaved, cleaned with povidone iodine, and a midline cervical incision was made from the level of larynx to the sternum. The submaxillary salivary glands were exposed by blunt dissection and separated through the midline fascial plane to expose the trachea. The left cervical vagus nerve, located within a neurovascular bundle with the left carotid artery lateral to the trachea, was separated from the surrounding tissue using fine forceps. The bundle is readily identified by the pulsation of the artery. The cervical vagus nerve, a white fiber travelling parallel to the carotid artery, was delicately separated from the artery using fine forceps (size 7). The vagus nerve was de-sheathed by gently removing the thin connective tissue surrounding the nerve under magnification using forceps (size 7S or 7). For recordings using hook electrode, the nerve was suspended on the hook away from the surrounding tissue and the surgical field bathed in mineral oil to both electrically insulate the nerve and prevent its desiccation. For recordings using cuff electrode, the cuff was submerged briefly in saline prior to nerve placement within the cuff.

### Recording procedure

The electrophysiological signals were digitized from the vagus nerve using either a Neuralynx data acquisition system (Digital Lynx 4SX, Cheetah v5 software, Neuralynx, Bozeman, MT) or a Plexon data acquisition system (Omniplex, Plexon Inc., Dallas, Texas). Recordings were sampled at 32 kHz and band-pass filtered between 10 and 9000 Hz for the Neuralynx, and 40 kHz with a 120 Hz filter and 50 gain for the Plexon. All signals were referenced to the animal ground placed between the right salivary gland and the skin. For recordings with three-lead hook electrodes, the signals from the most proximal lead were referenced with the most distal lead to minimize noise and improve the signal to noise ratio. The experimenter was always grounded while manipulating the animal during recordings. In experiments with LPS challenge, following acquisition of the baseline activity (10 min), 8.0 mg/kg ultra-pure LPS (Invitrogen, San Diego, California) was administered intraperitoneally, and recordings were continued for 10 min post-injection.

### Neurogram analysis

Vagus nerve recordings (termed “neurograms”) were analyzed using Spike2 software (Cambridge Electronics Design Limited, Cambridge, England). Raw recordings were filtered using a high-pass filter at 160 Hz followed by a “smoothing” algorithm consisting of a finite impulse response filter. Waveform analysis was done on the filtered recordings using a user-defined adaptive threshold method, and wave mark parameters (spike shape with a total spike time of 3 ms). Identified waveforms were manually categorized as neural spikes or other signals (cardiac, respiratory). The signals corresponding to cardiac and respiratory components were manually removed. Neural component with a CAP occurrence of >3X baseline was then analyzed to calculate rate and temporal distribution. Baseline and LPS neurograms were subjected to Fast Fourier Transform (FFT) to 0.0064 s with a Hanning window and 156.3 Hz resolution.

### Statistical analysis

Data are presented as individual samples, mean ± SD, and mean ± SEM. ANOVA, Student t test, and Mann-Whitney *U* test were used to examine for statistical significance. The variance within group was analyzed using Excel VAR function. *P* values < 0.05 were considered significant.

## Results

We developed an electrophysiological recording system to record compound action potentials transmitted in the cervical vagus nerve in mice, evaluated the performance of the electrophysiological recording system under various experimental conditions, and analyzed the recorded neural signals to identify stimulation specific neurogram patterns.

### Electrophysiological recording system for the cervical vagus nerve

Compound action potentials from the cervical vagus nerve were recorded using either a three-lead custom-built silver wire electrode (Fig. 1a) or a two-lead commercially available bipolar sling platinum-iridium cuff electrode (Fig. 1d) (CorTec, Germany). The silver wire leads are spaced 0.20 ± 0.04 cm apart and the ends are bent up into a hook shape. The leads in the cuff electrode with 200-μm inner diameter are flat and spaced approximately 0.10 cm apart. These electrodes are attached by solder or by cap and pin method to an electrode interface board (EIB). The electrode-EIB set-up is then attached to a head-stage that is wired to the acquisition system. For recording from the cervical vagus nerve, anesthetized mice are placed in a supine position. The vagus nerve is then exposed as described in the Methods; extreme care is taken to prevent any nerve trauma. The exposed nerve is placed over the three-lead silver hook electrode or a two-lead cuff electrode (Fig. 1c). A silver ground wire attached to the EIB is placed between the salivary gland and skin. In all recordings using the hook electrode, mineral oil was added to the surgical field to protect the vagus nerve from desiccation.

**Fig. 2:**
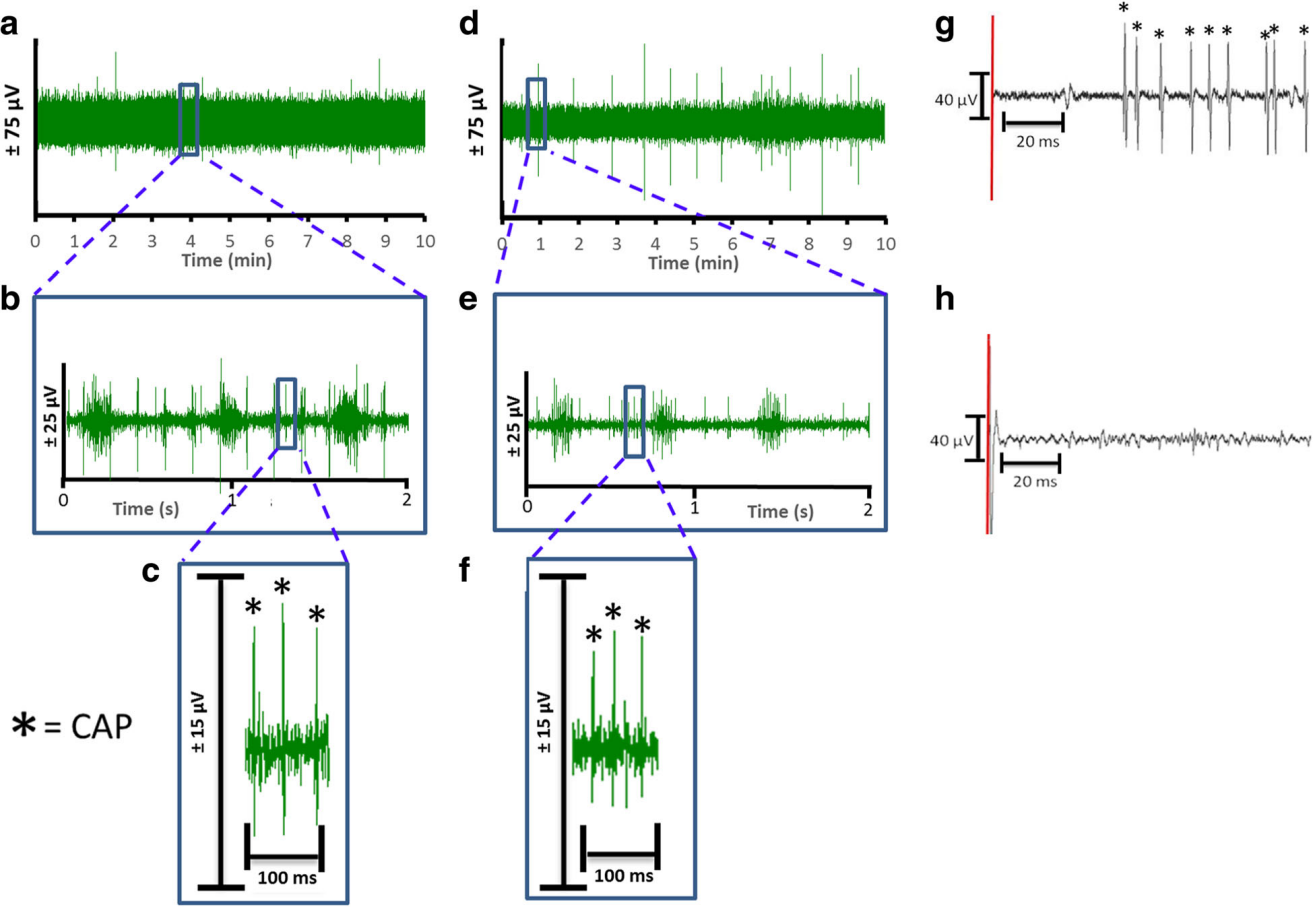
Comparison of neurograms recorded with hook and cuff electrodes. Baseline activity from the cervical vagus nerve was recorded for 10 min from Balb/c mice with either a hook (*n* = 72) or a cuff (*n* = 25) electrode. **a** Representative recording of the neural signals by three hook electrode in a 10 min window, **b**) The inset from (**a**) is expanded as a 2 s window, **c**) The inset from (**b**) is expanded as a 100 millisecond window with CAP’s indicated by the asterisk. **d** Representative recording of the neural signals recorded by the cuff electrode in a 10 min window, **e**) The inset from (**d**) is expanded as a 2 s window, and **f**) The inset from (**e**) is expanded as a 100 millisecond window with CAP’s indicated by the asterisk. **g** Addition of KCl (4 mM) indicated by the red line directly to the cervical vagus nerve causes depolarization and CAP’s to fire (indicated by asterisk). **h**) Pretreatment with 2% lidocaine inhibits the depolarization and subsequent CAP firing induced by the addition of KCl (4 mM) indicated by the red line

**Fig. 3:**
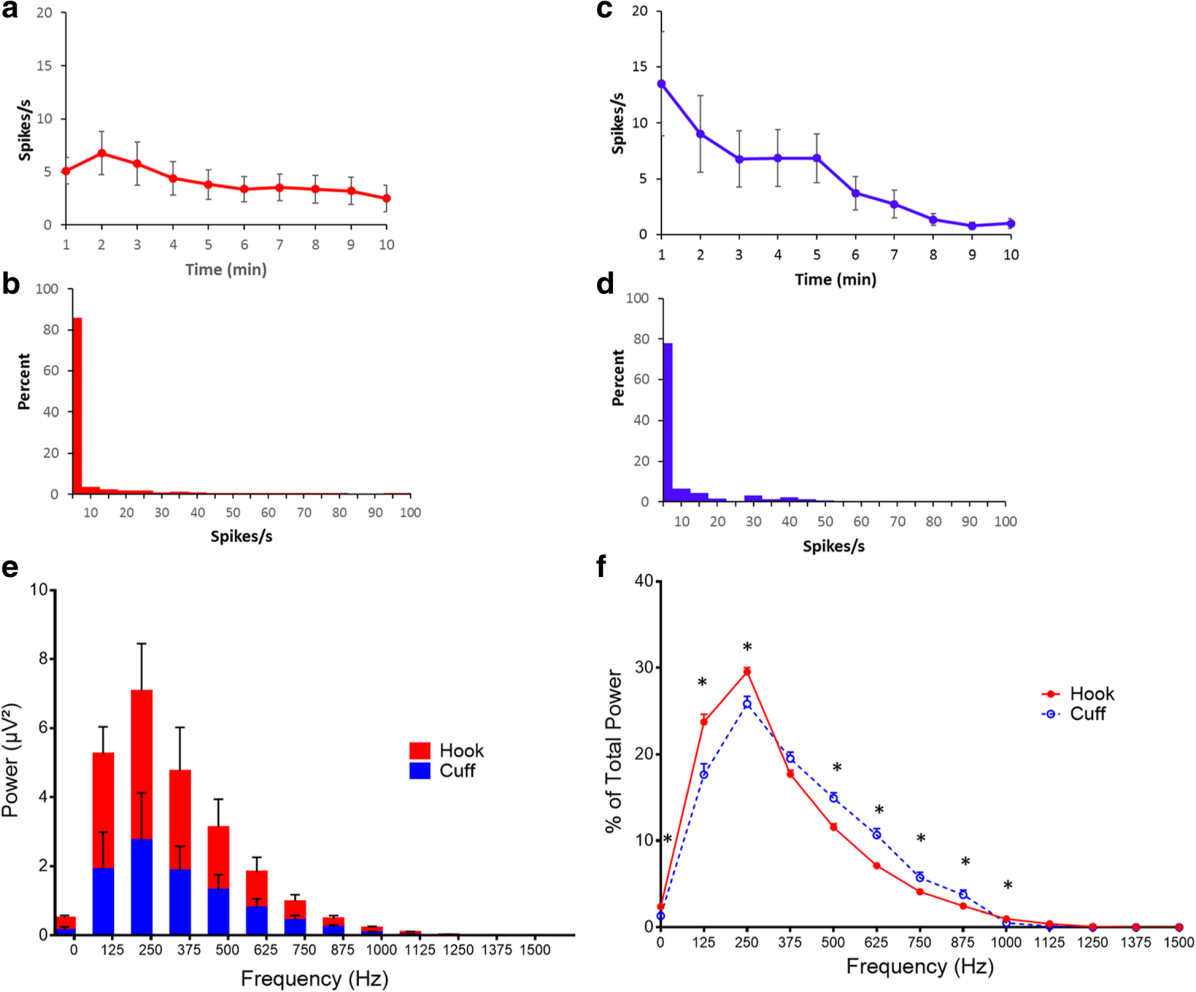
Comparison of neural activity recorded with hook and cuff electrodes. **a** Spikes rate of the neural events recorded with the hook electrode. **b** Rate distribution was calculated as overall percent of spike rate within 1 min segments over the 10-min recording with hook electrode. **c** Spikes rate of the neural events recorded with the cuff electrode. **d** Rate distribution was calculated as overall percent of spike rate within 1 min segments over the 10-min recording with cuff electrode. **e** FFT analysis of the 10 min recordings obtained with hook (red) or cuff (blue) electrode (Two-way ANOVA *p* = 0.0003). **f** Percentage breakdown of the frequencies recorded with the hook (red, closed circle solid line) and cuff (blue, open circle, dashed line)

Electrophysiological signals were recorded from the cervical vagus nerve using two different electrodes. The signals were acquired at either a 32 kHz and 40 kHz sampling rate (Neuralynx or Plexon respectively) and referenced to an animal ground electrode as described in methods. The electrical recording of compound action potential is obtained as the voltage tracings of the neural activity, and termed a neurogram (Fig. 2a and d, hook and cuff recordings respectively). Using a user-specified adaptive threshold method, action potential spikes are identified in the neurogram (Fig. 2c and f). Signals corresponding to noise, cardiac and respiratory components are removed to isolate the neural spikes within the recording (Fig. 2b and e). Neurograms recorded with three-lead hook electrode have an average spike rate of 4.2 ± 0.4 spikes/s for 10 min period (Fig. 3a). Analysis of 1 min segments revealed that more than 80% of the signal recorded with the three-lead hook electrode has a firing rate between 0 and 5 spikes/s (Fig. 3b). Recordings obtained using the two-lead cuff electrode has an average spike rate of 5.3 ± 0.8 spikes/s for 10 min period (Fig. 3c). Occurrence rate of 0–5 spikes/s over 80% of the recording was observed in cuff and hook recordings (Fig. 3b and d). We next analyzed the signal to noise ratio in hook and cuff electrodes. A power analysis of the entire neurogram after a 160 Hz high pass filter using Fast Fourier Transform showed a significantly higher amount of power in the hook recordings as compared to cuff recordings (Fig. 3e) (two-way ANOVA *p* = 0.0003). While comparing the frequency components of the recordings, a shift towards frequency components > 250 Hz in the cuff is observed (Fig. 3e and f). Percent of total power was significantly decreased at 0-250 Hz (Mann-Whitney, *p* < .005) in the cuff, while from 500 to 1000 Hz was significantly increased (Fig. 3f). This is further emphasized when identifying area under the curve, with a significant increase in power at > 250 Hz (Mann-Whitney, *p* < 0.0001, U = 476.5) in the cuff, as well as normalized power (two-way ANOVA, *p* < 0.0001) (data not shown).

### The effect of isoflurane on the baseline vagus nerve activity

**Fig. 4:**
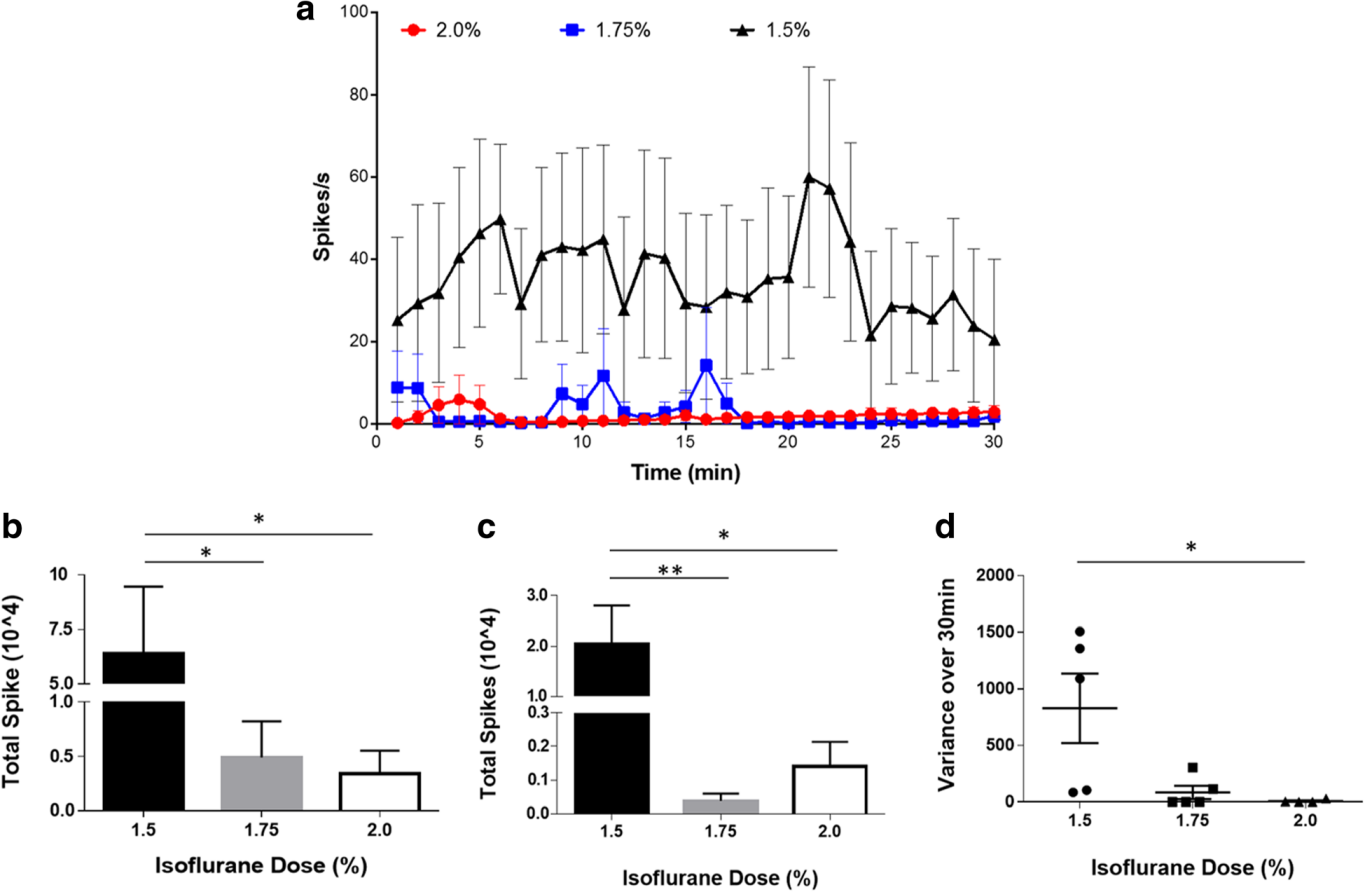
The effect of isoflurane on the baseline vagus nerve activity. Baseline vagus nerve activity was recorded for 30 min at different levels of isoflurane, 1.5% (*n* = 5), 1.75% (*n* = 5) and 2.0% (*n* = 4), using the cuff electrode. **a** Spikes rate of the neural events recorded for each isoflurane level. A significant difference in the baseline activity was found over three different levels of isoflurane (two-way ANOVA, *p* < 0.0001). **b** Total spike count over the entire 30-min recording. Total spikes with 1.5% isoflurane are significantly higher than 1.75% (Mann-Whitney, *p* < 0.05, U = 2) and 2% levels (Mann-Whitney, *p* < 0.05, U = 1). **c** Total spikes in the final 10 min of the recording. Total spikes with 1.5% isoflurane are significantly higher than 1.75% (Mann-Whitney, *p* < 0.01, U = 0) and 2% levels (Mann-Whitney, *p* <0 .05, U = 0). **d** The variance of the spike rate for each individual mouse over the 30-min recording period. Each dot represents variance in each mouse

The experimental protocol for acute vagus nerve recordings requires animals to be maintained under anesthesia for the duration of the procedure. In general inhalation anesthesia slows or blocks nerve impulses and affects synaptic transmission and neuronal function (Antkowiak, 1999; Antkowiak, 2001; Rudolph & Antkowiak, 2004). Isoflurane is a common choice of anesthetic due to its ease of use and easy monitoring during surgery. Here, we optimized the level of isoflurane that reduces background signals but did not significantly block spontaneous nerve activity. In these studies, anesthesia was maintained at three different levels of isoflurane (1.5%, 1.75% and 2.0%) with an oxygen flow rate of 1.0 L/min. To determine the optimal isoflurane level that allows a consistent low firing rate (0–10 spikes/s) the neurogram data is analyzed using Spike 2 analysis software. A significant difference in 30 min neurograms was found between the three different levels of isoflurane (Two-way ANOVA, *p* < 0.0001). Higher variability over 30-min recording period was observed at a lower level of isoflurane (1.5%) with a range of 0–127 spikes/s (Fig. 4a). Increasing levels of isoflurane induced a dose-dependent suppression of the baseline activity with an average spike rate over the thirty-minute recording of 35.5 ± 3.6 spikes/s for 1.5% of isoflurane (*n* = 5), 2.7 ± 0.8 spikes/s for 1.75% (*n* = 5), and 1.9 ± 0.3 spikes/s for 2.0% (*n* = 5) (Fig. 4a). Thus, there is a significant decrease in total spikes over the 30-min recording period with higher anesthesia levels (Fig. 4b). Next, we analyzed the spike rates for the 10-min neurogram. The low level of isoflurane (1.5%) resulted in higher baseline activity (34.1 ± 6.2 spikes/s) in the 10-min period as compared to 1.75% (0.6 ± 0.2 spikes/s) and 2% levels (2.3 ± 0.4 spikes/s). In addition, significantly higher total spikes are observed at 1.5% isoflurane compared to 1.75% and 2% isoflurane levels (Fig. 4c). Analysis of the variance of the spike rate for each individual mouse over the 30-min recording period (Fig. 4d) revealed that the recordings obtained at 1.5% isoflurane level have a significantly higher variance as compared to 1.75% and 2% levels. Moreover, with lower level of isoflurane (1.5%), the animals have high variance within the group as compared to higher levels of isoflurane. Together, this data demonstrates that the 1.5% of isoflurane fails to ablate the background noise activity level whereas the highest level of isoflurane studied, 2%, blunted the neural response almost completely. In contrast, 1.75% isoflurane produced anesthesia while enabling a low level of baseline activity (2.7 ± 0.8 spikes/s).

### The effect of food intake on the baseline vagus nerve activity

**Fig. 5:**
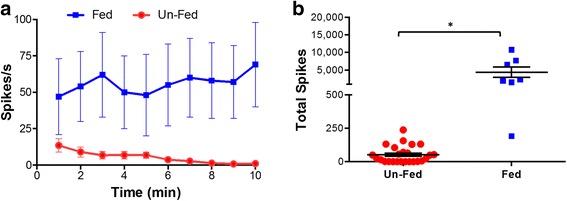
The effect of food intake on the baseline vagus nerve activity. **a** Plot of fed mice (*n* = 8, solid line, closed circles) and un-fed mice (*n* = 25, dashed line open boxes) shows significant differences at all time points (*p* < 0.05). **b** Total spike count over the entire 10-min recording in un-fed and fed mice (T-test *p<* 0 .0001)

The vagus nerve is the main conduit of a bidirectional communication between the central nervous system and the gut. The rich innervation of the gastrointestinal tract by both sensory and motor fibers of the vagus nerve is essential for normal homeostasis, and plays an important role in regulating food intake, digestion, GI barrier function and immunity (Berthoud & Neuhuber, 2000; Berthoud et al., 1991; Stakenborg et al., 2013). To evaluate the effects of the food intake and digestion on the acute vagus nerve activity, we recorded vagus nerve activity in two groups of mice. One group, the ‘fed-group’, was allowed to have access to food till the time of the experiment (*n* = 8). For the other group, the ‘un-fed group’, food was removed 3 to 4 h prior to recording (*n* = 25). Significantly higher baseline vagus nerve activity was observed over 10-min recordings in the ‘fed-group’ mice that were allowed to have access to food prior to recordings compared to the un-fed group (*p* < 0.05, unpaired t-test) (Fig. 5a). The average spike rate over 10-min period in fed mice was significantly higher (55.9 ± 2.1 spikes/s, *p* < 0.05, unpaired t-test) compared to the fasted mice (5.3 ± 0.8 spikes/s). Significantly higher numbers of total spikes over this interval was also observed in the fed group compared to the un-fed group (fed animals: 33,533.8 ± 10,129 spikes, un-fed animals: 7,043 ± 794 spikes; *p* < 0.05, Mann-Whitney test) (Fig. 5b). Together, these findings indicate that food intake significantly modulates vagus neurograms in mice.

### Baseline vagus nerve activity is comparable between different mouse strains

**Fig. 6:**
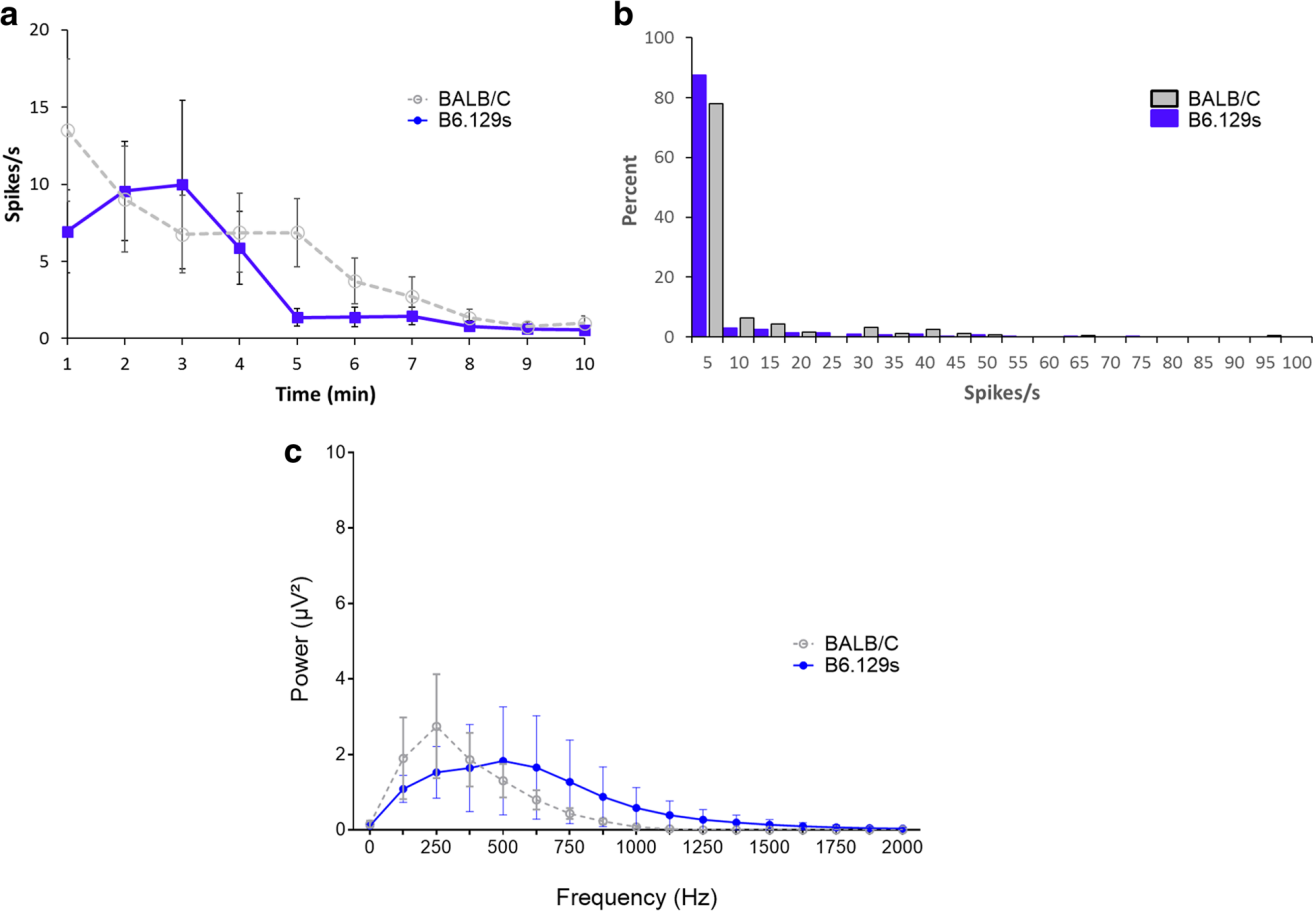
Baseline vagus nerve activity is comparable between different mouse strains. Baseline vagus nerve activity was recorded in C57BL/6.129S (*n* = 31) and Balb/c mice (*n* = 25) with a cuff electrode. **a** Spikes rate of the neural events recorded. **b** Rate distribution in 1 min segments over 10-min recording. **c** FFT analysis of the 10 min recordings. No significant difference between different mouse strains was observed (two-way ANOVA, *p* = 0.51)

C57BL/6 and Balb/c strains are among the most widely studied mouse strains in immunology, neuroscience, electrophysiology and experimental disease research. C57BL/6 and Balb/c mice differ as Th1-type and Th2-type mouse strains, respectively, and respond differentially to innate immune challenges (Kyuwa et al., 2003; Watanabe et al., 2004). Balb/c strain is often used as an experimental model of acute inflammation whereas C57BL/6.129S (B6.129S) strain is usually used as a background in the generation of knockout and transgenic animals. To determine if baseline vagus nerve activity differs between these strains, we recorded vagus neurograms in B6.129S and Balb/c mice using a cuff electrode. The average spike rate over the period of 10-min baseline recording in B6.129S mice is 3.9 ± 0.8 spikes/s (Fig. 6a, *n* = 31) which is comparable to the average spike rate of Balb/c mice 5.3 ± 0.8 spikes/s (Fig. 3b, *n* = 25) (*p* = 0.53, U = 349, Mann Witney T-test). Most vagus nerve activity (80%) in B6.129S and Balb/c mice is between 0 and 5 spikes/s at baseline (Fig. 6b). No significant difference in the total power of the 10-min signal recorded using cuff electrode is observed between Balb/c and B6.129S strains (Fig. 6c, *p* = 0.51, two-way ANOVA). Together, this data indicates that there is no significant strain specific difference in baseline activity of the vagus nerve in murine Balb/c and B6.129S strains.

### LPS induces increased rate of cervical vagus nerve activity

**Fig. 7:**
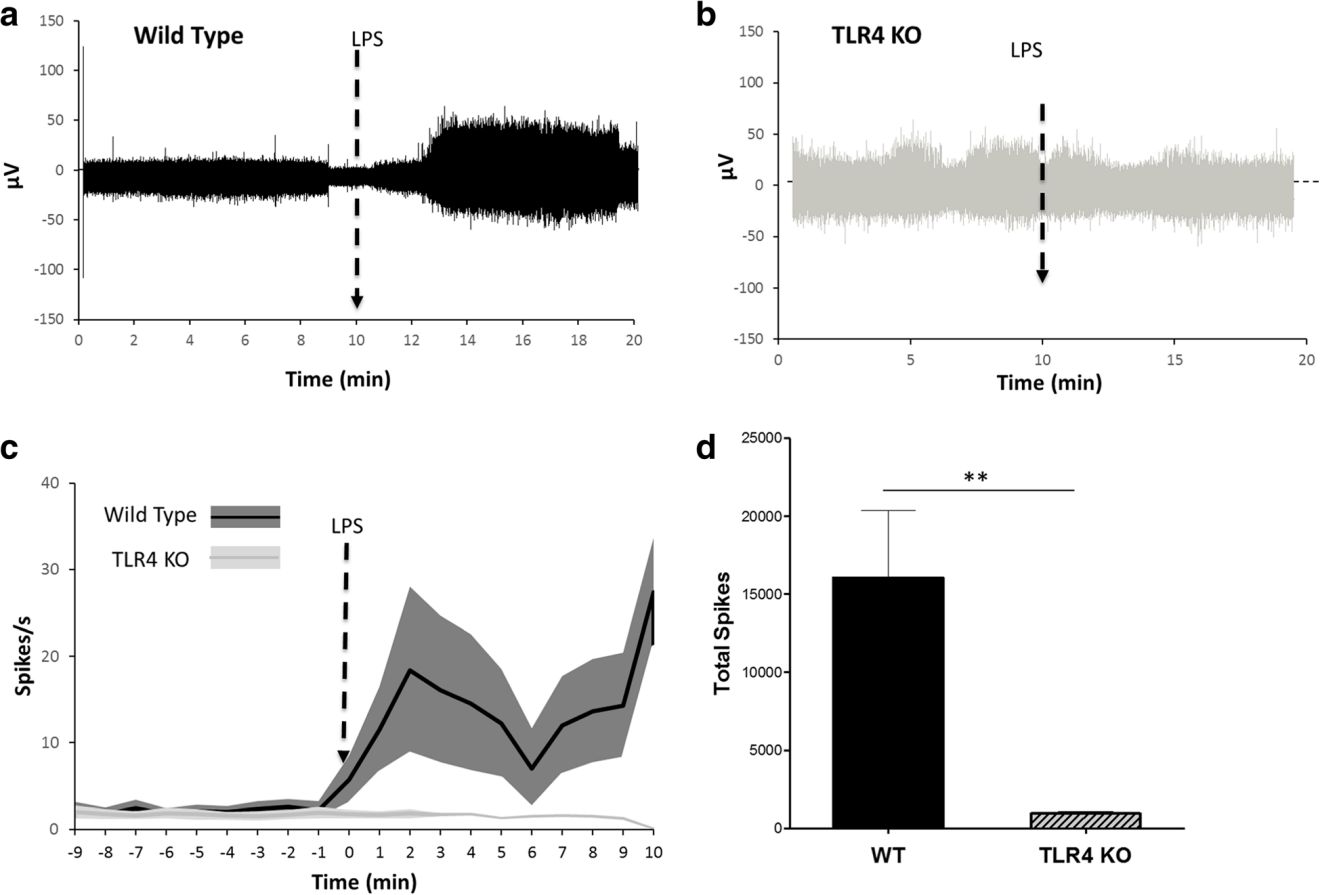
Time course of LPS-induced neurogram in wild type and TLR4 KO mice. Representative neurogram in **a**) wild type and **b**) TLR4 KO mice. The arrow represents the time of ip injection of 8.0 mg/kg LPS. **c** Spikes rate of the neural events recorded in wild type (*n* = 13) and TLR4 KO (*n* = 4). There is a significant difference between the post injection responses between the TLR4 KO and wild type (t test, *p* < 0.0001). **d** Total spike count over the entire 10-min recording is significantly different between wild type and TLR4 KO mice (Mann-Whitney, *p* < 0.001, U = 00)

We next evaluated the changes in neural activity associated with inflammatory stimuli by recording vagus nerve activity in response to LPS administration. LPS is a cell-wall component of the gram-negative bacteria, and administration of LPS leads to induction of a robust inflammatory response in a toll-like receptor 4 (TLR4)-dependent manner (Nijland et al., 2014; Lu, 2008; Poltorak et al., 1998). Changes in vagus nerve activity were recorded in wild type mice as described in the methods. After recording 10 min of baseline activity, animals received intraperitoneal administration of LPS. Injection of LPS lead to a significant increase in vagus nerve activity (26.7 ± 3.6 spikes/s) as compared to baseline activity (4.1 ± 0.4 spikes/s, *p* < 0.0001, T = 7.67, Mann-Whitney test) (Fig. 7a and c). Vagus neurograms from TLR4 KO mice failed to exhibit changes (1.6 ± 0.1 spikes/s) as compared to baseline activity (1.8 ± 0.3 spikes/s, *p* > 0.99, U = 8, Mann-Whitney test) (Fig. 7b and c). Total spike count over the entire 10-min recording after LPS administration is significantly less in TLR4 KO mice compared to wild type mice (Fig. 7d) (Mann-Whitney, *p* < 0.001, U = 00). Together, these data provide direct evidence that LPS-TLR4 ligand-receptor interaction is required for inducing changes in vagus nerve activity during endotoxemia.

## Discussion

Here, we have established and evaluated a method for recording compound action potentials of the murine cervical vagus nerve in real time. By optimizing the experimental conditions to reduce the magnitude of the baseline activity, the method enables recording of the changes in vagus nerve activity in response to exogenous challenges. Multiple factors may affect baseline vagus nerve activity including electrode design, degree and type of anesthesia, gut functions, and genetic background of the mouse strains. Although the spike events in the recordings obtained with hook electrode and cuff electrode are similar, the power analysis of the recorded signal provides clear evidence that the cuff electrodes offer a better signal to noise ratio (Fig. 3e and f). There is ample evidence that cuff electrodes offer a stable platform for peripheral nerve signal recording (Sahin & Durand, 1998; Dweiri et al., 2015; Durand, 2007).

Inhalation anesthetics have profoundly suppressive effects on neuronal function. Isoflurane is commonly used for surgical procedures due to its multiple positive features. Induction of isoflurane anesthesia results in less stress, the doses can be monitored or modified during procedure, and in general isoflurane is easy to use. However, isoflurane may lead to immunomodulatory effects in the experimental models of vagus nerve stimulation (Picq et al., n.d.). Importantly, isoflurane induces a dose-dependent inhibitory effect on nerve activity, nerve conduction velocity and neural network functions (Detsch et al., 2002; Picker et al., 2001; Skovsted & Sapthavichaikul, 1977; Lv et al., 2016; Malinowsky et al., 1995; Sellgren et al., 1994). Here, we compared the effect of increasing doses of isoflurane on baseline activity of vagus nerve. Baseline vagus nerve activity at 1.5% is very variable over time. In contrast, the nerve activity is significantly suppressed at 2%. This is in accordance to the previous observations that isoflurane exhibited a dose dependent change in the renal nerve activity with a significant suppression of nerve activity at 2.5% isoflurane as compared to both control and 1.5% isoflurane (Seagard et al., 1984). In contrast, a low level of baseline activity was observed at 1.75% isoflurane indicating an optimal dose of isoflurane that can be used for recording vagus nerve signals without blunting the neural response. Studies with another inhalation anesthetic, halothane, showed that increasing halothane levels from 1% to 4% decreased both hypoglossal nerve and phrenic nerve activities in a dose-related manner. However, halothane had a distinct temporal response on hypoglossal nerve and phrenic nerve, suggesting that respiratory control of the tongue muscles and the diaphragm are in part mediated by different neural pathways (Nishino et al., 1984). Moreover, type of anesthetic further determines the level of suppression of nerve activities. Thiopental and diazepam but not ketamine induces a dose-dependent differential suppression of hypoglossal nerve and phrenic nerve activities (Nishino et al., 1984). Together, these findings emphasize the importance of selecting an appropriate anesthetic and titrating the dose of anesthetic for individual experimental set-up for recording nerve activity.

The vagus nerve is the primary sensory nerve in the gut-brain axis, transmitting signals related to food intake to the central nervous system, and play a vital role in the feedback loop controlling food intake (Schwartz, 2000). Electrophysiological recording studies have identified mechanoreceptors, chemoreceptors, osmoreceptors and temperature receptors in the gut (Berthoud & Powley, 1992; Cummings & Overduin, 2007; Lal et al., 2001; Moriarty et al., 1997; Buyse et al., 2001). The sensory vagus nerve detects various food related signals and transmits information concerning levels of lipids, cholecystokinin, leptin, peptide YY, insulin and glucose to the brain (Yi et al., 2010) in real time leading to appropriate efferent output from the dorsal motor nucleus. These motor signals are transmitted via the efferent vagus nerve to the gastrointestinal tract, liver and pancreas and modulate metabolic and dietary function (Stakenborg et al., 2013; Yi et al., 2010). Our studies clearly demonstrate that animals with access to food prior to the recording procedure have active baseline vagus nerve activity that is diminished when animals are fasted prior to neural recording (Fig. 5). Gastrointestinal vagus nerve afferents are involved in the regulation of short-term feeding behavior (Yi et al., 2010; Owyang & Heldsinger, 2011). Cholecystokinin is secreted from small intestinal I cells in response to food ingestion (Polak et al., 1975; Buchan et al., 1978) and function as a postprandial satiety signal (Weller et al., 1990; Crawley & Corwin, 1994). Electrophysiological studies have provided evidence that vagus nerve afferents mediate signals related to cholecystokinin to the brainstem (Li & Owyang, 1993; Blackshaw & Grundy, 1990). Food intake also regulates effects of leptin on afferent vagus nerve activity (Kentish et al., 2013). Both cholecystokinin and leptin activate vagus afferents synergistically and mediate an efferent vagus response leading to inhibitory effects on food intake (Owyang & Heldsinger, 2011). Together these studies provide clear evidence that food intake prior to nerve recordings will influence both the afferent and efferent baseline vagus nerve activity. In experiments designed to identify activation signals in the vagus nerve in response to exogenous challenge, it is therefore important to not feed the animals prior to collecting neurograms in order to minimize baseline vagus nerve activity.

Mice are the most widely-used species used as the experimental models of human diseases due to the availability of the immunological and molecular reagents as well as transgenic and knockout models. Further, genetic analysis has revealed that mouse genome shares high degree of homology with the human genome (Mural et al., 2002; Chinwalla et al., 2002). The availability of inbred strains further offers a maximum genetic uniformity. However, strain specific differences have been reported for phenotypic, behavior, stress-induced and immunological responses in mice strains (Van Bogaert et al., 2006; Cramer et al., 2015; Marques et al., 2011; De Vooght et al., 2010; Crawley et al., 1997). Spontaneous rhythmic electroencephalographic (EEG) activity, a hallmark of the central nervous system activity, varies significantly in different mouse strains (Franken et al., 1998). Circadian rhythms analysis in inbred mouse strains further demonstrate significant differences between Balb/c and C57BL/6 mice (Schwartz & Zimmerman, 1990). Strain-specific differences in neural serotonergic pathways have also been observed in Balb/c and C57BL/6 mice (Neal et al., 2009). Further, naturally occurring variability in anesthetic potency have been demonstrated in different mouse strains (Sonner et al., 2000). However, the differences in vagus neurograms in two commonly used mouse strains, Balb/c and C57BL/6 had not been previously characterized. Here, our studies clearly show that Balb/c and B6.129S vagus neurograms are comparable. Although the baseline vagus nerve activity is comparable it remained possible that the induced vagus nerve activity in response to inflammatory challenges may differ in these two strains. Specifically, Balb/c and C57BL/6 mice exhibit Th2 type and Th1 type phenotype respectively and respond differentially to stress (Cramer et al., 2015; Savignac et al., 2011), bacterial clearance (Watanabe et al., 2004) and various immunological challenges (Watanabe et al., 2004; Marques et al., 2011; De Vooght et al., 2010; Barone et al., 1993; Gueders et al., 2009). Future work will determine if there are strain-specific differential changes in induced vagus nerve activity in response to different inflammatory conditions.

We have recently demonstrated that the cytokine induced vagus nerve activity can be recorded in real time. By electrically stimulating or suppressing the vagus nerve activity with lidocaine or tetrodotoxin, we have confirmed that recorded vagus nerve activity is a function of neuronal activity (Steinberg et al., 2016). Here, we have established methodologies for recording stable baseline vagus nerve activity, and recording vagus nerve activity in real time in response to exogenous challenges such as lipopolysaccharide, a bacterial endotoxin. Our previous studies using surgical vagotomies have verified that the majority of the signals recorded on the cervical vagus nerve are mediated by the afferent fibers (Steinberg et al., 2016) that relay inflammation specific signals to the brain. To study the receptor dependency of LPS-induced responses, we used LPS-receptor TLR4 knock out (TLR4 KO) mice. Wild type mice but not the TLR4 KO mice showed enhancement in the vagus neurograms in response to LPS indicating that TLR4 receptor-LPS interaction is required to mediate the neurogram response. These studies corroborate the previous findings that vagus afferent neurons express TLR4, and that LPS can activate afferent vagus nerve activity (Hosoi et al., 2005). Together, these methods for recording vagus neurograms in mice provide a useful tool to further delineate the role of vagus neural pathways in different murine disease models.

## Conclusion

Vagus afferents innervating the visceral organs including pancreas, liver, gut provide a rapid and discrete account of the changes in the physiological conditions in real time. In addition, the vagus nerve also senses and transmits information about the inflammatory phenotype of the host to the central nervous system. Selective activation of the vagus nerve in response to different challenges suggests the intriguing possibility that vagus neurograms might serve as a monitoring system for delineating the host’s inflammatory status in real time. Currently, there are many devices that modulate electrical impulses in the vagus nerve to treat rheumatoid arthritis, Crohn’s and epilepsy. However, these devices are dependent on external monitoring and modulation to alter the stimulation paradigm. This study establishes methodology for recording vagus nerve activity in real time at baseline condition and in response to exogenous challenges. A detailed mapping of the neural responses in real time could enable development of a ‘close-loop’ system that can record sensory vagus nerve activity and physiological parameters, analyze the data in real time and modulate the electrical stimulation and neural activity accordingly.
